# Impact of lack-of-benefit stopping rules on treatment effect estimates of two-arm multi-stage (TAMS) trials with time to event outcome

**DOI:** 10.1186/1745-6215-14-23

**Published:** 2013-01-23

**Authors:** Babak Choodari-Oskooei, Mahesh KB Parmar, Patrick Royston, Jack Bowden

**Affiliations:** 1 London Hub for Trials Methodology Research, MRC Clinical Trials Unit, Aviation House, 125 Kingsway, WC2B 6NH, London

## Abstract

**Background:**

In 2011, Royston *et al.* described technical details of a two-arm, multi-stage (TAMS) design. The design enables a trial to be stopped part-way through recruitment if the accumulating data suggests a lack of benefit of the experimental arm. Such interim decisions can be made using data on an available ‘intermediate’ outcome. At the conclusion of the trial, the definitive outcome is analyzed. Typical intermediate and definitive outcomes in cancer might be progression-free and overall survival, respectively. In TAMS designs, the stopping rule applied at the interim stage(s) affects the sampling distribution of the treatment effect estimator, potentially inducing bias that needs addressing.

**Methods:**

We quantified the bias in the treatment effect estimator in TAMS trials according to the size of the treatment effect and for different designs. We also retrospectively ‘redesigned’ completed cancer trials as TAMS trials and used the bootstrap to quantify bias.

**Results:**

In trials in which the experimental treatment is better than the control and which continue to their planned end, the bias in the estimate of treatment effect is small and of no practical importance. In trials stopped for lack of benefit at an interim stage, the treatment effect estimate is biased at the time of interim assessment. This bias is markedly reduced by further patient follow-up and reanalysis at the planned ‘end’ of the trial.

**Conclusions:**

Provided that all patients in a TAMS trial are followed up to the planned end of the trial, the bias in the estimated treatment effect is of no practical importance. Bias correction is then unnecessary.

## Background

The two-arm, multi-stage (TAMS) trial design described by Royston *et al.*[1] provides a framework for efficiently evaluating an experimental treatment regimen against a control group, by using an intermediate outcome to potentially cease the trial for lack of benefit at an early stage. Choosing appropriate and valid intermediate (*I*) and definitive (*D*) outcomes is key to the success of a TAMS trial, for which Royston *et al.*[1] provides guidance. In this framework, we assume that both the intermediate and final outcomes are time-to-event outcomes. The basic assumptions are that *I* occurs no later than *D*, more frequently than *D* and is on the causal pathway to *D*. If the null hypothesis is true for *I*, it must also hold for *D*. In the absence of an obvious choice for *I*, a rational choice of *I* might be *D* itself earlier in time. In this instance, of course, *I* does not occur more frequently than *D*. The TAMS design framework can be well suited to cancer trials. In the cancer context, typical intermediate and definitive outcomes might be progression-free survival (PFS) and overall survival (OS), respectively. Information on PFS is usually available sooner in a study, and in most cancer sites, the treatment effect on PFS is usually highly positively correlated with that on OS [1].

It is well known that stopping a trial early, for example in sequential and group sequential designs, may yield biased estimates of the treatment effect (Piantadosi [2], pp. 183, 387). By the ‘treatment effect’ we mean the difference on some suitable scale between the experimental and control arms; typically, for time-to-event data this will be the (log) hazard ratio between the survival distributions under proportional hazards (PH). When a trial is stopped early because accumulating evidence favors the alternative hypothesis, the maximum partial likelihood estimate (MPLE) of the treatment effect – in the context of the Cox PH model – is biased in the direction of the alternative hypothesis. The earlier a trial is stopped, the larger the potential bias [2]. Although the TAMS design framework can help (and is helping [3]) to expedite the discovery and evaluation of new and effective treatments, concerns have been raised about possible bias in the final treatment effect estimate induced by this approach, for example, hazard ratios (*HR*_*D*_) on OS for trials with time-to-event outcomes.

In a TAMS trial, recruitment is halted at one of the interim stages if there is insufficient evidence in favor of the alternative hypothesis. Emerson [4] showed that applying any stopping rule affects the sampling distribution of the MPLE of the treatment effect (see Figure 3 in [4]) and consequently induces a potential bias. The distribution of test statistics and their *P* values are similarly affected by such rules. However, hypothesis testing is not the focus of the present paper as it has been already addressed in [1]. Bias is present in the estimated treatment effect whether or not a trial is stopped for lack of benefit. However, bias in treatment effect estimates in trials passing all interim lack-of-benefit assessments is more important than that in stopped trials, since such experimental treatments are much more likely to be considered worthy of further study or adoption into clinical practice.

Regardless of an interim decision on whether to stop or not, it is still important to estimate the treatment effect using all available data. Royston *et al.*’s [5] proposal to terminate recruitment of new patients if the experimental arm fails to show evidence of benefit, while at the same time continuing to follow up all recruited patients, was designed to make TAMS trials cost efficient but also to mitigate possible bias. However, the precise magnitude of the bias present in the final treatment effect estimate has not been rigorously explored. In this paper, we investigate the bias in the estimates of treatment effects resulting from a TAMS design. We also define the ‘selection bias’ in estimated hazard ratios and empirically quantify its likely magnitude in TAMS trials through simulation studies and bootstrap-based reanalyses of four completed cancer trials.

The structure of the paper is as follows. In the Methods section, we first outline how a TAMS trial is specified, noting the required design parameters and assumptions. Next, we discuss the ‘selection’ bias induced in TAMS trials by the use of lack-of-benefit stopping guidelines. For simplicity, we discuss this issue in a two-stage TAMS setting. We describe our simulation study intended to explore the magnitude of the bias. The simulation study is carried out in a three-stage TAMS setting. In this section, we also introduce four real trials and ‘redesign’ them as if they were TAMS trials. In the Results, we present simulation results. We also show the results of our bootstrap reanalyses of the example trials in an empirical assessment of bias at the definitive analysis of the treatment effect. This is followed by a discussion.

## Methods

### Specification of a TAMS design

In a TAMS trial, we compare one experimental arm, *E*, with a control arm, *C*. A TAMS design has *s* ≥ 2 stages. The first *s* − 1 stages assess lack of benefit by comparing *E* with *C* on an intermediate outcome, *I*. The *s*th stage compares *E* with *C* for efficacy on the definitive outcome, *D*. Let *HR*_*I*_ be the underlying hazard ratio for comparing *E* with *C* on *I*, and *HR*_*D*_be the underlying hazard ratio comparing *E* with *C* on *D*.

We assume that proportional hazards hold between the treatment arms, and also that the times to event are exponentially distributed for both *I* and *D* outcomes, with control-arm hazard rates of *λ*_*I*_ and *λ*_*D*_, respectively.

The null and alternative hypotheses for a TAMS design are:

$$\begin{array}{ll} H_{0}\;\text{at stages}\;1\;\text{to}\;s-1 & \;:\;HR_{I}=HR_{I}^{0}\\ H_{1}\;\text{at stages}\;1\;\text{to}\;s-1 & \;:\;HR_{I}=HR_{I}^{1}\\ \;H_{0}\;\text{at stage}\;s & \;:\;HR_{D}=HR_{D}^{0}\\ \;H_{1}\;\text{at stage}\;s & \;:\;HR_{D}=HR_{D}^{1} \end{array}$$

The primary null and alternative hypotheses, *H*_0_ (stage *s*) and *H*_1_ (stage *s*), concern *HR*_*D*_, with the hypotheses on *I* playing a subsidiary role. However, we require design values for all the hypotheses. In practice, $HR_{I}^{0}$ and $HR_{D}^{0}$ are almost always taken as 1. In cancer trials, $HR_{D}^{1}=0.75$ is a common choice.

Taking $HR_{I}^{1}=HR_{D}^{1}$ is a conservative option; the design allows for the possibility that $HR_{I}^{1}<HR_{D}^{1}$. For example, in cancer, if *I* is the earlier of progression or death and *D* is death, it may be realistic and efficient to take, say, $HR_{D}^{1}=0.75$ and $HR_{I}^{1}=0.7$.

By definition, if *E* is better than *C* then $HR_{I}<HR_{I}^{0}$ and $HR_{D}<HR_{D}^{0}$. Let ${\hat{\Delta }}_{i}$ (*i* < *s*) be the estimated hazard ratio comparing *E* with *C* on outcome *I* for all patients recruited up to and including stage *i*, and ${\hat{\Delta }}_{s}$ be the hazard ratio comparing *E* with *C* on *D* for all patients at stage *s* (that is, at the time of the analysis of the definitive outcome). The design is specified as follows:

Applies to all stages:

1. Define the hazard rates *λ*_*I*_and *λ*_*D*_, or equivalently, the median times to event.

2. Define hazard ratios $HR_{I}^{0}$, $HR_{I}^{1}$, $HR_{D}^{0}$ and $HR_{D}^{1}$. Usually, $HR_{I}^{0}=HR_{D}^{0}=1$.

3. Define the allocation ratio, *A*, that is the number of patients allocated to *E* for every patient allocated to *C*. *A* = 1 represents equal allocation; with *A* < 1 relatively fewer patients are allocated to *E*, and with *A* > 1, relatively more patients are allocated to *E*.

**For stages 1 to***s* − 1**:**

1. For stage *i*, define a one-sided significance level *α*_*i*_and power *ω*_*i*_. The motivation for one-sided tests is that we are interested only in rejecting the null hypothesis in the direction of benefit of *E* over *C*, not harm. We also specify *r*_*i*_, the expected total patient accrual rate per unit time.

2. From these inputs, the nstage software [6] reports *e*_*i*_, the cumulative number of events to be observed in the control arm during stages 1 through *i*; *n*_*i*_, the number of patients to be entered in the control arm during stage *i*; *An*_*i*_, the corresponding number of patients in the experimental arm; *t*_*i*_, the approximate (calendar) time, *t*_*i*_, of the end of stage *i*, under the design assumptions; and a critical value, *δ*_*i*_, for rejecting $H_{0}:HR_{I}=HR_{I}^{0}$.

3. If ${\hat{\Delta }}_{i}\geq \delta_{i}$, the null hypothesis of $HR_{I}=HR_{I}^{0}$ cannot be rejected at the *α*_*i*_level, and the trial is stopped for lack of benefit of *E* over *C*. Otherwise, ${\hat{\Delta }}_{i}<\delta_{i}$, suggesting some degree of benefit of *E*, and recruitment continues to the next stage.

**Stage***s*:

The same principles apply to stage *s* as to stages 1 to *s* − 1. Here, *e*_*s*_ is the required number of control arm events for the *D* outcome, cumulative over all stages. We would typically recommend a one-sided significance level of *α*_*s*_ = 0.025 at stage *s*, corresponding to a conventional two-sided 0.05 level.

If the treatment comparison survives all of the *s* − 1 tests at step 3 above, the trial proceeds to the final stage, otherwise recruitment is terminated early. Mathematical details of the sample size calculations are given in Section Methods of Royston *et al.*[1].

### Interim selection on a definitive outcome

Here we consider the bias induced in the estimated treatment effect in a two-stage TAMS design with *I* = *D*. A lack-of-benefit stopping rule is applied at the first (interim) stage. If the treatment comparison shows some evidence of benefit of the experimental arm, recruitment continues and the definitive analysis is performed at a later second stage. Otherwise, recruitment is terminated.

Let *θ*_*D*_ be the underlying log hazard ratio (log *HR*) comparing the experimental treatment with control. We define *θ*_*D*_ such that *θ*_*D*_ < 0 denotes a true advantage of the experimental treatment over control. Let ${\hat{\theta }}_{D}$ be the MPLE of *θ*_*D*_. In the absence of stopping rules, ${\hat{\theta }}_{D}$ is asymptotically unbiased and approximately normally distributed in repeated realizations of the trial ( [7], p. 40). No bias enters, and so:

(1) $$E\left[{\hat{\theta }}_{D}\right]=\theta_{D}$$

over repeated realizations of the trial.

Let ${\hat{\theta }}_{D}$ be the estimated log *HR* for the data accumulated at the first stage (lack-of-benefit analysis). Recruitment stops if ${\hat{\theta }}_{D}\geq \log(\delta )$ and continues to the final stage if ${\hat{\theta }}_{D}<\log(\delta )$. The threshold *δ*is predefined according to a designated significance level and power. We have:

(2) $$E\left[{\hat{\theta }}_{D}|{\hat{\theta }}_{D}<\log(\delta )\right]=\theta_{D}-B_{1}$$

(3) $$E[{\hat{\theta }}_{D}|{\hat{\theta }}_{D}\geq \log(\delta )]=\theta_{D}+B_{2}$$

where *B*_1_*B*_2_ > 0 and are functions of *θ*_*D*_. *B*_1_ and *B*_2_ may be termed the selection bias [8] in ${\hat{\theta }}_{D}$ in the two scenarios. Expressions 2 and 3 state that under the PH assumption ${\hat{\theta }}_{D}$ is biased downwards by *B*_1_ and upwards by *B*_2_ in continuing and stopped trials, respectively.

**Figure 1:**
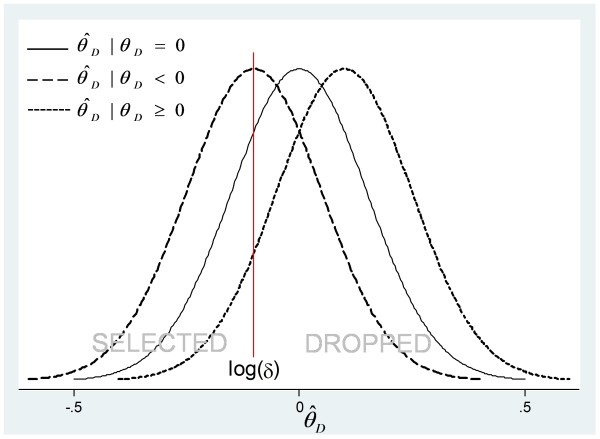
**Sampling distribution of **${\hat{\theta }}_{D}$**, that is, estimated log hazard ratios, which are normally distributed, under different underlying effects, *θ***_***D***_**. ***δ* is the predefined threshold.

As an illustration, Figure 1 shows hypothetical sampling distributions (densities) of ${\hat{\theta }}_{D}$ at the first stage for treatments with *θ*_*D*_ negative, zero or positive. The vertical line denotes a typical lack-of-benefit threshold, log(*δ*) < 0. The probability of passing the lack-of-benefit threshold, that is $\text{Pr}\;\left[{\hat{\theta }}_{D}<\log(\delta )\right]$, is the area under the appropriate density to the left of *δ*. Trials of the treatment for which *θ*_*D*_ < 0 (long-dashed line) have the largest chance of passing, and those for which *θ*_*D*_ > 0 (short-dashed line) have the largest chance of stopping.

Selection bias *B*_1_ among ‘passed trials’ is the largest for the treatment with *θ*_*D*_ > 0 and smallest for that with *θ*_*D*_ < 0. Conversely, selection bias *B*_2_ among ‘stopped trials’ is the largest for the treatment with *θ*_*D*_ > 0 (dotted line) and smallest for that with *θ*_*D*_ < 0 (dashed line).

### Interim selection on an intermediate outcome

**Figure 2:**
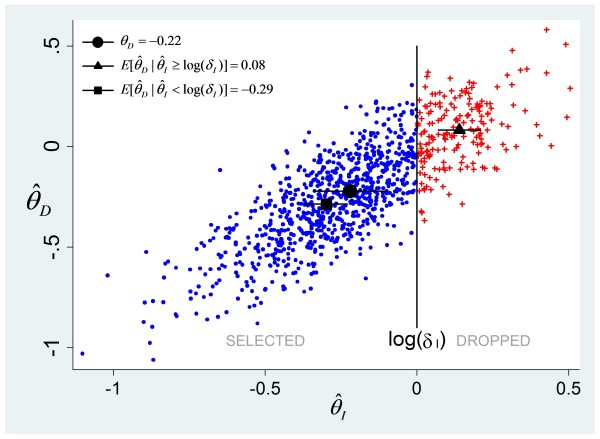
**Log hazard ratio pairs of (PFS, OS) simulated from a bivariate normal distribution with mean (log(0.8), log(0.8)) and a correlation coefficient of 0.8. ***δ*_*I*_ is the predefined threshold. Blue circles and red crosses represent selected and dropped trials, respectively. OS, overall survival, PFS, progress-free survival.

We now consider the more complex scenario where we use a different outcome *I* at the interim stage. In Royston *et al.*’s [1] TAMS design it was proposed to cease/continue accrual according to the value of an intermediate outcome measure that is correlated with the definitive outcome measure. An example is selection on the basis of PFS log *HR*s but ultimately estimating the OS log *HR*. Now let ${\hat{\theta }}_{D}$ and ${\hat{\theta }}_{I}$ be treatment effect estimates on the *D* and *I* outcomes, respectively, at the interim stage. The selection bias in ${\hat{\theta }}_{D}$ given that ${\hat{\theta }}_{I}$ passed the predefined threshold $\log(\delta_{I})$ could be expressed as:

(4) $$E\left[{\hat{\theta }}_{D}|{\hat{\theta }}_{I}<\log\left(\delta_{I}\right)\right]=\theta_{D}-B_{3}$$

for some *B*_3_ that depends on the underlying values *θ*_*D*_, *θ*_*I*_ and their correlation, $\rho_{\theta_{I},\theta_{D}}$. To illustrate this we assume, as in Royston *et al.*[1], that ${\hat{\theta }}_{I}$ and ${\hat{\theta }}_{D}$ follow a bivariate normal distribution with correlation $\rho_{\theta_{I},\theta_{D}}$. Figure 2 shows 1,000 log hazard ratio pairs, ( ${\hat{\theta }}_{I},{\hat{\theta }}_{D}$), simulated from a bivariate normal distribution with mean (log(0.8),log(0.8)) and a correlation coefficient of 0.8. Dots represent values of ( ${\hat{\theta }}_{I},{\hat{\theta }}_{D}$) in simulated trials in which ${\hat{\theta }}_{I}<\delta_{I}$. Because ${\hat{\theta }}_{I}$ and ${\hat{\theta }}_{D}$ are correlated, it is clear that the mean of ${\hat{\theta }}_{D}$ in trials that either continue ( ${\hat{\theta }}_{I}<\log(\delta_{I})$) or are stopped ( ${\hat{\theta }}_{I}\geq \log(\delta_{I})$) is biased with respect to *θ*_*D*_. In this example, the selection bias of the ‘stopped’ trials is larger than that of the ‘continuing’ trials, since the square is closer to the circle than the triangle is to the circle.

### Simulation study

We conducted simulation studies to quantify the impact of various stopping rules on the estimates of the *θ*_*D*_, that is log(*HR*_*D*_), in three-stage TAMS designs with two interim analyses. We considered bias in two situations: (i) simulated trials with an underlying hazard ratio close to the null hypothesis, which are likely to be stopped at the first of the two intermediate stages due to apparent lack of efficacy; (ii) simulated trials with an underlying hazard ratio close to the alternative hypothesis, which are more likely to pass both intermediate stages to reach the final stage (analysis of the *D* outcome). To fix ideas, we took the *D* outcome as OS and the *I* outcome (used for selection) as either OS or PFS. We denote the OS hazard ratio and PFS hazard ratio as *HR*_*D*_ and *HR*_*I*_, respectively. When the *I* outcome was PFS, we generated correlated PFS and OS times to event according to the method of Royston *et al*. [1].

Design parameter values were based on the GOG182/ICON5 trial in advanced ovarian cancer [9]. We assumed the median time-to-event for OS and PFS outcomes to be 2 years and 1 year, respectively. (When I=D, we assumed the median time-to-event to be 1 year.) For sample size calculations, we chose the target hazard ratio to be 0.75 for efficacy and 1.0 for inefficacy on both outcome measures at all stages. Note that when *I* ≠ *D*, TAMS designs allow the target hazard ratio(s) for efficacy at intermediate stages to be different (for example, more extreme) than the target hazard ratio at the final stage. Such designs would be even more efficient, but we adopted the conservative option of taking all target hazard ratios for efficacy to be the same across stages.

We considered two TAMS designs (1 and 2) defined by different sets of significance levels (*α*_1_, *α*_2_, *α*_3_) at the three stages. We took (*α*_1_, *α*_2_, *α*_3_) = (0.5, 0.25, 0.025) in Design 1 and (0.2, 0.1, 0.025) in Design 2. The interim analyses in Design 1 take place earlier than those in Design 2. As suggested by Royston *et al*. [1], we designated a stage-specific power (*ω*_1_, *ω*_2_, *ω*_3_) = (0.95, 0.95, 0.90) in both designs. Table 1 gives details of the design parameters. Calculations for Table 1 were done in Stata using the program nstage[6].

**Table 1:** **Design parameters for two three-stage TAMS**^**a **^**trials**

						***I *and *D *outcomes: OS**^**b**^					***I *outcomes: PFS**^**c**^**; *D *outcomes: OS**^**b**^			
**Design**	**Stage**	***α***_***i***_^**d**^	***ω***_***i***_^**e**^	***r***_***i***_^**f**^	***δ***_***i***_^**g**^	***e***_***i***_^**h**^	***t***_***i***_^**i**^	***N***_***i***_^**j**^	***HR***^**1**^^**k**^		***e***_***i***_^**h**^	***t***_***i***_^**i**^	***N***_***i***_^**j**^	***HR***^**1**^^**k**^
1	1	0.50	0.95	250	1.00	73	1.53	382	0.75		73	1.53	382	0.75
	2	0.25	0.95	250	0.92	140	2.62	566	0.75		140	2.62	566	0.75
	3	0.025	0.90	250	0.84	262	3.40	851	0.75		264	4.36	1091	0.75
2	1	0.2	0.95	250	0.91	159	2.45	612	0.75		159	2.45	612	0.75
	2	0.1	0.95	250	0.89	217	3.00	750	0.75		217	3.00	750	0.75
	3	0.025	0.90	250	0.84	262	3.40	851	0.75		264	4.36	1091	0.75

^a^Two-arm multi-stage;

^b^overall survival;

^c^progression-free survival;

^d^nominal significance level at stage *i*;

^e^nominal power at stage *i*;

^f^rate of patient accrual per unit time during stage *i*;

^g^predefined threshold;

^h^cumulative number of control arm events required at end of stage *i*;

^i^duration (in time units) up to the end of stage *i*;

^j^cumulative number of patients accrued to control arm by end of stage *i*;

^k^target hazard ratio under ***H***_***1***_, the target hazard ratio for inefficacy ***HR***^***0***^is 1 in all scenarios.

When generating simulated times to event, we applied each of four underlying hazard ratios: 1.1 and 1.0, to represent trials with an ineffective experimental treatment, and 0.88 and 0.75, to represent trials with an effective experimental treatment. The first two represent situation (i), whereas the latter two represent situation (ii) as explained above. In our simulations, 5,000 trials were replicated in each experimental condition. For trials which stopped at stage 1, we computed the mean of estimated OS log hazard ratios, that is $\bar{\log(HR_{D})}$, at that stage. For trials that reach the final stage, $\bar{\log(HR_{D})}$ is computed at that stage. In all scenarios, we report the results on the hazard ratio scale. To provide an estimate of spread, we also present the 2.5th and 97.5th centiles of the estimated OS hazard ratios. Aside from hazard ratios, we also report the absolute value (size) of percentage bias which is defined as:

(5) $$\%\;\text{Bias}=100\times \frac{\left(\text{Estimated}\;\text{HR}-\text{Underlying}\;\text{HR}\right)}{\text{Underlying}\;\text{HR}}$$

Data were simulated with staggered patient entry at a uniform accrual rate of 250 individuals per year. Equal numbers of patients were allocated to control and experimental arms in all stages. We also carried out similar simulations with target hazard ratios for efficacy of 0.85 instead of 0.75, requiring larger numbers of *I* and *D* events and generally longer timelines.

### Bootstrap reanalysis

#### Trials used as examples

To evaluate selection bias in the estimated treatment effects, we also ‘reanalyzed’ the data from four MRC-coordinated cancer trials as though the trials were run as two-stage TAMS designs (that is one interim analysis). The selected trials comprise two in advanced renal cancer (RE01 [10], RE04 [11]) and two in advanced ovarian cancer (ICON3 [12], ICON4 [13]). All except for RE04 were also reanalyzed from a methodological perspective by Barthel *et al.*[14]. ICON3 and RE04 were ‘unsuccessful’ in that no conventionally statistically significant treatment effect was found. ICON4 and RE01 were conventionally ‘successful’ and demonstrated clear evidence of improvement in survival due to the experimental therapy. Some details of the trial results are given in Table 2.

**Table 2:** The estimated PFS and OS hazard ratios at the end of the four example trials

**Trial**	**Control arm**			**Experimental arm**			**Hazard ratio (95% CI)**			
	**Treatment**	***N***^**a**^	***e***^**b**^	**Treatment**	***N***^**a**^	***e***^**b**^	**PFS**^**c**^	***P *value**	**OS**^**d**^	***P *value**
ICON3	Carbo/CAP	1350	827	Carbo, TAX	698	431	0.93(0.83–1.03)	0.15	0.97(0.86–1.09)	0.63
RE04	IFN-*α*	502	340	IFN-*α*, IL-2,5FU	504	351	1.02(0.89–1.16)	0.81	1.05(0.90–1.21)	0.55
ICON4	Plat.	378	220	Plat., TAX	361	199	0.81(0.69–0.95)	0.01	0.82(0.68–0.99)	0.04
RE01	MPA	176	167	IFN-*α*	174	155	0.68(0.54–0.84)	<0.01	0.75(0.60–0.93)	<0.01

^a^Number of patients;

^b^overall survival events, that is deaths from any cause;

^c^progression-free survival;

^d^overall survival.

**Figure 3:**
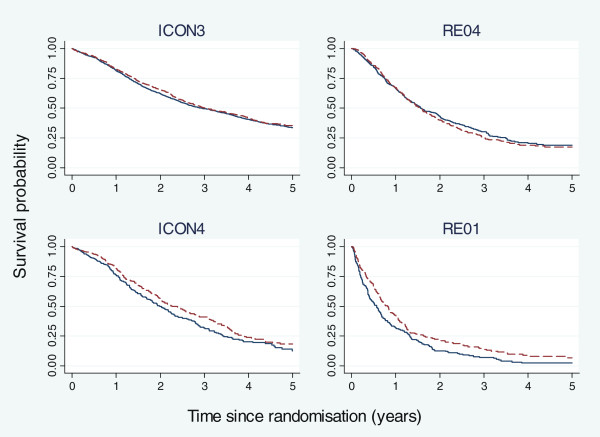
**Kaplan–Meier plots of overall survival in four example trials, truncated at 5 years.** The solid curve represents the control arm, and the dashed curve represents the experimental arm in all four graphs.

Figure 3 shows the Kaplan–Meier plots of OS in the trials, truncated at 5 years. There is a suspicion in Figure 3 that the survival curves of the two treatment groups may cross in the RE04 trial, suggesting possible non-proportional hazards. However, this was not confirmed by Grambsch–Therneau tests [15].

#### Design

We ‘redesigned’ all four example trials as two-stage TAMS designs. Design parameters are given in Table 3 and are based on values in the original trial protocols. We used the nstage program [6] to compute the required number of control arm events for the *I* outcome at stage 1 (interim analysis for lack of efficacy) and the *D* outcome at stage 2 (final analysis of the definitive outcome). We took the *D* outcome to be OS, and the *I* outcome to be OS or PFS in separate analyses. We studied five one-sided significance levels *α*_1_ = (0.1, 0.2, 0.3, 0.4, 0.5) at stage 1, providing progressively earlier looks at the accumulating data. Stage 2 one-sided significance level was *α*_2_ = 0.025 in all the scenarios.

**Table 3:** Parameter values for trial reanalysis based on trial protocols

**Design parameter**	**Unsuccessful trials**		**Successful trials**	
	**ICON3**	**RE04**	**ICON4**	**RE01**
$HR_{I}^{1}$ and $HR_{D}^{1}$	0.75	0.80	0.75	0.71
$HR_{I}^{0}$ and $HR_{D}^{0}$	1.0	1.0	1.0	1.0
Power at stage 1 (*ω*_1_)	0.95	0.95	0.95	0.95
Power at stage 2 (*ω*_2_)	0.90	0.90	0.90	0.90
Overall power	0.855	0.855	0.855	0.855
Allocation ratio (control : experimental)	2:1	1:1	1:1	1:1
Median time to event for *I* outcome (months)	18	5.5	10	2.5
Median time to event for *D* outcome (months)	36	12	23	10

We ‘entered’ patients one by one in the same order as they had presented in the original trial. Stage 1 analysis was conducted when the target number of *I* events had accrued (see Tables 4 and 5). Patients who had not entered by the time of the stage 1 analysis were excluded from the interim analysis. Trials were ‘stopped’ for lack of efficacy at stage 1 or continue recruitment to the final analysis at stage 2.

**Table 4:** Simulation results for the trials which stop at stage 1

		$HR_{D}^{1}=0.75$					$HR_{D}^{1}=0.85$					
**Design**	**Under-**	**%Stop**	**Estimated *HR***_***D ***_**for trials stopped at stage 1**					**%Stop**	** Estimated *HR***_***D ***_**for trials stopped at stage 1**			
	**lying**	**at**	**At interim point**		**After follow-up**			**at**	**At interim point**		**After follow-up**	
	***HR***_***D***_	**stage 1**	**Mean (centiles**^**a**^**)**	**%Bias**	**Mean (centiles**^**a**^**)**	**%Bias**		**stage 1**	**Mean (centiles**^**a**^**)**	**%Bias**	**Mean (centiles**^**a**^**)**	**%Bias**
I outcome: OS^b^												
1	1.10	70	1.19(1.01,1.52)	8	1.14(0.95,1.39)	4		83	1.13(1.01,1.33)	3	1.12(0.99,1.28)	2
	1.00	50	1.14(1.00,1.42)	14	1.06(0.89,1.29)	6		50	1.08(1.00,1.24)	8	1.05(0.93,1.18)	5
	0.88	22	1.10(1.00,1.32)	25	0.98(0.81,1.19)	11		10	1.04(1.00,1.16)	18	0.97(0.88,1.08)	10
	0.75	4	1.06(1.00,1.23)	41	0.89(0.75,1.09)	19		0.2	1.03(1.00,1.12)	37	0.90(0.82,1.03)	20
2	1.10	95	1.11(0.93,1.36)	1	1.11(0.94,1.32)	1		99	1.10(0.98,1.24)	0	1.10(0.99,1.22)	0
	1.00	80	1.04(0.92,1.25)	4	1.03(0.89,121)	3		80	1.02(0.95,1.14)	2	1.02(0.94,1.13)	2
	0.88	37	0.98(0.91,1.13)	11	0.95(0.84,1.09)	8		13	0.98(0.95,1.05)	11	0.95(0.90,1.03)	8
	0.75	5	0.95(0.91,1.05)	27	0.89(0.80,1.00)	19		0	−	−	−	−
I outcome: PFS^c^												
1	1.10	71	1.17(0.81,1.68)	6	1.11(0.92,1.38)	1		83	1.13(0.92,1.41)	3	1.11(0.98,1.29)	1
	1.00	51	1.12(0.79,1.59)	12	1.05(0.87,1.28)	5		50	1.07(0.88,1.31)	7	1.04(0.91,1.19)	4
	0.88	22	1.06(0.77,1.53)	20	0.96(0.80,1.17)	9		9	1.01(0.83,1.19)	15	0.95(0.84,1.09)	8
	0.75	4	1.03(0.72,1.44)	37	0.88(0.71,1.04)	17		0	−		−	
2	1.10	96	1.11(0.85,1.44)	1	1.09(0.93,1.30)	1		99	1.10(0.95,1.28)	0	1.10(0.99,1.24)	0
	1.00	79	1.04(0.81,1.34)	4	1.02(0.87,1.20)	2		79	1.02(0.90,1.16)	2	1.01(0.92,1.13)	1
	0.88	39	0.97(0.76,1.23)	10	0.94(0.81,1.09)	7		13	0.96(0.85,1.07)	9	0.94(0.86,1.04)	7
	0.75	4	0.92(0.74,1.13)	23	0.87(0.75,0.99)	16		0	−	−	−	−

^a^The 2.5th and 97.5th centiles of the estimated OS hazard ratios;

^b^overall survival;

^c^progression-free survival.

**Table 5:** Simulation results for the trials that reach the final stage

**Design**	**Under-**	$HR_{D}^{1}=0.75$			$HR_{D}^{1}=0.85$		
	**lying**	**Estimated *HR***_***D ***_**for trials reached final stage**			**Estimated *HR***_***D ***_**for trials reached final stage**		
	***HR***_***D***_	**%Pass**^**a**^	**Mean (centiles**^**b**^**)**	**%Bias**	**%Pass**^**a**^	**Mean (centiles**^**b**^**)**	**%Bias**
I outcome: OS^c^							
1	1.10	7	0.97(0.85,1.11)	− 12	2	1.01(0.94,1.10)	− 8
	1.00	21	0.92(0.80,1.05)	− 8	22	0.95(0.88,1.03)	− 5
	0.88	61	0.85(0.73,0.98)	− 3	84	0.87(0.79,0.95)	− 1
	0.75	94	0.74(0.62,0.88)	− 1	99.9	0.75(0.68,0.83)	0
2	1.10	1	0.90(0.78,1.01)	− 18	0	−	−
	1.00	10	0.86(0.79,0.98)	− 14	9	0.92(0.87,0.97)	− 8
	0.88	48	0.82(0.71,0.92)	− 7	81	0.87(0.79,0.93)	− 1
	0.75	93	0.74(0.62,0.86)	− 1	99.9	0.75(0.68,0.83)	0
I outcome: PFS^d^							
1	1.10	7	1.01(0.86,1.17)	− 8	2	1.04(0.96,1.13)	− 5
	1.00	22	0.94(0.80,1.09)	− 6	23	0.97(0.88,1.05)	− 3
	0.88	61	0.85(0.73,0.99)	− 3	84	0.87(0.79,0.96)	− 1
	0.75	93	0.74(0.62,0.88)	− 1	99.8	0.75(0.68,0.83)	0
2	1.10	1	0.97(0.82,1.11)	− 12	0	−	−
	1.00	9	0.91(0.79,1.05)	− 9	9	0.95(0.86,1.03)	− 5
	0.88	47	0.84(0.72,0.98)	− 5	82	0.87(0.79,0.95)	− 1
	0.75	93	0.74(0.62,0.88)	− 1	100	0.75(0.68,0.83)	0

^a^Percentages of trials that pass interim stages 1 and 2 and continue accrual to the final stage;

^b^the 2.5th and 97.5th centiles of the estimated OS hazard ratios;

^c^overall survival;

^d^progression-free survival.

Similar to our simulation studies, the number of replicates was 5,000 in our bootstrap analysis of example trials. In each replicate, the two types of selection bias (in stopped ‘unsuccessful’ trials, and in ‘successful’ trials) were investigated exactly as in the simulation study. Means of OS log hazard ratios at stage 1 and at the planned end of the trials are calculated separately. In all scenarios, we report the results on the hazard ratio scale.

## Results

### Simulation results

The simulation results are summarized in Tables 4 and 5. Table 4 gives the results for the trials that stop at stage 1. The percentage of simulated trials in which the estimated log hazard ratio exceeds the stage 1 threshold log(*δ*_1_) is given. This is identified by ‘%Stop at stage 1’ in Table 4. According to the TAMS design, recruitment to such trials is ceased at the interim stage. Table 4 presents the average treatment effect on the *D* outcome, that is *HR*_*D*_, together with the 2.5th and 97.5th centiles for the trials that stopped at stage 1. We also followed up the individuals in the same stopped trials to the original planned end and computed the estimates of OS hazard ratios then. Table 4 also shows the average of treatment effect on the *D* outcome at the end of the follow-up period in those trials that stopped at stage 1 for lack of efficacy.

The average treatment effect for trials stopped at stage 1 is biased in all experimental conditions. This bias increases as the underlying hazard ratio moves from 1.1 to 0.75. However, the smaller the underlying hazard ratio, the less likely a trial is to stop at stage 1 – see %Stop in Table 4. The bias is smaller in Design 2 because the lower significance level in stage 1 increases the required number of events and makes the data more mature at this point than in Design 1.

The results in Table 4 indicate that the true (underlying) hazard ratio is overestimated at stage 1 in all scenarios. A key finding is that when follow-up of patients in stopped trials is continued to the planned end of the final stage, the bias is much reduced. For instance, in Design 1 when the target hazard ratio $HR_{D}^{1}=0.75$ with an underlying *HR*_*D*_ of 1.1 – the first row in the left panel of Table 4 – the percentage bias in the average treatment effect for the trials which stopped at stage 1 is 8% – that is 100 × (1.19 − 1.10) / 1.10. This decreases to 4% after follow-up to the planned end of the trial. In all cases, after follow-up of patients to the original planned end of the trial, the bias is generally minimal (mostly less than 6%) if the underlying effect is in the direction of null hypothesis. In the dropped trials, the bias is slightly smaller when the intermediate outcome, that is PFS, is used for selection at the interim stages.

We also calculated the average of treatment effect on the *D* outcome for trials that stopped at either of the two interim stages (data not shown). The selection bias is very similar to the corresponding values in Table 4 when the *I* outcome is PFS, and the bias becomes smaller when the *I* outcome is OS.

Table 5 presents the average treatment effect on the *D* outcome at the final stage for the trials that pass both interim stages. The bias at the final stage is generally smaller when the target hazard ratio $HR_{D}^{1}=0.85$, compared with the corresponding values when target hazard ratio $HR_{D}^{1}=0.75$. However, the main result from this table is that in the trials that reach the final stage, the selection bias in the average treatment effect is very small provided that the underlying effect is closer to the alternative hypothesis. There is some bias in the average treatment effect when the underlying effect is closer to the null hypothesis, but in such scenarios the chance that the research arm is dropped at the interim stages is large – see %Pass in Table 5.

### Bootstrap results

The results of the bootstrap reanalyses of the trials showing evidence of an effect (ICON3 and RE04) are summarized in Tables 6 and 7. Table 6 shows the average treatment effect on the *D* outcome for the trials that stopped at stage 1 together with their corresponding 2.5th and 97.5th centiles. For the left side of the table OS was used at the interim stage to select trials. On the right PFS was used at the interim stage to select trials. The number of replications was 5,000 in all experimental conditions.

**Table 6:** Bootstrap results for the stopped trials based on ICON3 and RE04 in a two-stage design

		***I *and *D *outcomes are OS**^**a**^						***I *outcome is PFS**^**b**^**, *D *outcome is OS**^**a**^					
			**%Stop**	**Estimated *HR***_***D ***_**for trials stopped at stage 1**					**%Stop**	**Estimated *HR***_***D ***_**for trials stopped at stage 1**			
			**at**	**At interim point**		**After follow-up**			**at**	**At interim point**		**After follow-up**	
***α***_**1**_^**c**^	***δ***_**1**_^**d**^	***e***_**1**_^e^	**stage 1**	**Mean (centiles)**	**%Bias**	**Mean (centile)**	**%Bias**	***e***_**1**_^**e**^	**stage 1**	**Mean (centiles)**	**%Bias**	**Mean (centiles)**	**%Bias**
a) ICON3 –		*HR*_*D*_(95% bootstrap CI): 0.97(0.87–1.09)											
0.50	1.00	118	38	1.12(1.00,1.40)	15	0.96(0.84,1.10)	1	116	55	1.27(0.88,1.83)	31	0.93(0.79,1.10)	− 4
0.40	0.97	153	55	1.08(0.97,1.31)	11	0.98(0.86,1.11)	1	152	52	1.15(0.83,1.56)	19	0.95(0.81,1.10)	− 2
0.30	0.94	195	52	1.03(0.94,1.22)	6	0.98(0.87,1.10)	1	194	33	1.09(0.81,1.45)	12	0.97(0.85,1.11)	0
0.20	0.91	252	42	0.99(0.91,1.15)	2	0.99(0.89,1.10)	2	250	12	1.16(0.89,1.46)	20	1.00(0.89,1.12)	3
0.10	0.89	342	44	0.95(0.89,1.08)	− 2	0.99(0.90,1.10)	2	339	29	1.06(0.86,1.31)	9	1.00(0.90,1.12)	3
b) RE04 –		*HR*_*D*_(95% bootstrap CI): 1.05(0.90–1.21)											
0.50	1.00	118	34	1.09(1.00,1.27)	4	1.10(0.95,1.26)	5	116	33	0.92(0.66,1.32)	− 12	1.07(0.89,1.30)	2
0.40	0.97	155	36	1.06(0.97,1.26)	1	1.09(0.96,1.24)	4	152	67	0.89(0.67,1.19)	− 15	1.02(0.86,1.23)	−3
0.30	0.95	199	71	1.05(0.95,1.23)	0	1.05(0.93,1.20)	0	196	79	0.95(0.74,1.23)	− 10	1.05(0.90,1.24)	0
0.20	0.93	259	84	1.05(0.95,1.23)	0	1.06(0.94,1.21)	1	255	73	0.96(0.78,1.19)	− 9	1.04(0.90,1.21)	− 1
0.10	0.91	355	79	1.05(0.92,1.26)	0	1.06(0.92,1.22)	1	351	97	1.02(0.86,1.22)	− 3	1.03(0.89,1.20)	− 2

^a^Overall survival;

^b^progression-free survival;

^c^one-sided significance level at stage 1;

^d^predefined threshold at stage 1;

^e^cumulative number of control arm events required at end of stage 1.

**Table 7:** Bootstrap results for the trials that reach the final stage based on ICON3 and RE04

		***I *and *D *outcomes are OS**^**a**^				***I *outcome is PFS**^**b**^**, *D *outcome is OS**^**a**^			
		**Estimated *HR***_***D ***_**for trials reached final stage**				**Estimated *HR***_***D ***_**for trials reached final stage**			
***α***_**1**_^**c**^	***δ***_**1**_^**d**^	***e***_**1**_^**e**^	**%Pass**^**f**^	**Mean (centiles)**	**%Bias**	***e***_**1**_^**e**^	**%Pass**^**f**^	**Mean (centiles)**	**%Bias**
b) RE04 –		*HR*_*D*_(95% bootstrap CI): 1.05(0.90–1.21)							
0.50	1.00	118	62	0.96(0.86,1.07)	− 1	116	45	0.96(0.85,1.07)	− 1
0.40	0.97	153	45	0.97(0.85,1.06)	0	152	48	0.95(0.85,1.07)	− 2
0.30	0.94	195	48	0.95(0.85,1.06)	− 2	194	67	0.96(0.86,1.07)	− 1
0.20	0.91	252	58	0.95(0.85,1.06)	− 2	250	88	0.97(0.86,1.08)	0
0.10	0.89	342	56	0.95(0.85,1.05)	− 2	339	71	0.96(0.86,1.06)	− 1
b) RE04 –		*HR*_*D*_(95% bootstrap CI): 1.05(0.90–1.21)							
0.50	1.00	118	66	1.02(0.89,1.16)	- 3	116	67	1.03(0.89,1.19)	− 2
0.40	0.97	155	64	1.02(0.89,1.16)	- 3	152	33	1.01(0.88,1.16)	−4
0.30	0.95	199	29	0.98(0.87,1.10)	−7	196	21	0.99(0.87,1.13)	−6
0.20	0.93	259	16	0.95(0.85,1.04)	−10	255	27	0.99(0.87,1.13)	−6
0.10	0.91	355	21	0.97(0.86,1.08)	−8	351	3	0.94(0.80,1.07)	−10

^a^Overall survival events;

^b^progression-free survival;

^c^one-sided significance level at stage 1;

^d^predefined threshold at stage 1;

^e^cumulative number of control arm events required at end of stage 1;

^f^percentages of trials that pass the interim stage and continue accrual to the final stage.

Results in Table 6 indicates that bias is present in the trials that did not pass stage 1. For example, the original OS hazard ratio in ICON3 is 0.97 (95% bootstrap CI: 0.87–1.09). For this trial, 1907 (38%) of the 5,000 replicated trials stopped at stage 1 when *I* = *D* and *α*_1_ = 0.50 – the first line in the left panel of Table 6. The average treatment effect for the stopped trials is 1.12. But, after the follow-up, the average treatment effect reduces to 0.96 and the selection bias nearly disappears. In general, the bias decreases with decreasing *α*_1_ and *δ*_1_. This is due, as before, to the increasing amount of information (that is patient events) that is required at the first interim for these design parameters. In all example trials, the bias is very small after the follow-up if the stage 1 significance level *α*_1_ is chosen to be smaller than 0.40.

Furthermore, for the scenarios presented in Table 6, we also computed the average treatment effect on the *D* outcome at the final stage for the trials which passed the interim stage. The results, presented in Table 7, show that the bias in the average treatment effect in those trials is very small in most scenarios. Results for RE04 show some bias in some scenarios, but the chance of passing the interim stage is relatively small in those conditions – see %Pass.

The results of the bootstrap reanalyses of the ‘successful’ trials (ICON4 and RE01) are summarized in Tables 8 and 9. Table 8 shows the results for the trials that reach the final stage. For ICON4, 99% of trials reached the final stage when *α*_1_ = 0.5 and the *I* outcome was OS. In contrast to the unsuccessful trials, the results for the successful trials show that there is almost no bias in the estimated hazard ratio on OS at the final stage.

**Table 8:** Bootstrap results for trials that reached the final stage based on ICON4 and RE01

		***I *and *D *outcomes are OS**^**a**^				***I *outcome is PFS**^**b**^**, *D *outcome is OS**^**a**^			
		**Estimated *HR***_***D ***_**for trials reached final stage**				**Estimated *HR***_***D ***_**for trials reached final stage**			
***α***_**1**_^**c**^	***δ***_**1**_^**d**^	***e***_**1**_^**e**^	**%Pass**^**f**^	**Mean (centiles)**	**%Bias**	***e***_**1**_^**e**^	**%Pass**^**f**^	**Mean (centiles)**	**%Bias**
a) ICON4 –		*HR*_*D*_(95% bootstrap CI): 0.82(0.67,0.99)							
0.50	1.00	75	99	0.82(0.67,0.98)	0	75	99	0.82(0.67,0.99)	0
0.40	0.96	98	97	0.82(0.67,0.97)	0	97	99	0.82(0.67,0.99)	0
0.30	0.94	126	96	0.82(0.67,0.97)	0	125	98	0.82(0.67,0.98)	0
0.20	0.91	163	92	0.81(0.67,0.94)	− 1	162	96	0.81(0.67,0.97)	1
0.10	0.89	222	86	0.81(0.67,0.94)	− 1	221	89	0.81(0.67,0.96)	1
b) RE01 –		*HR*_*D*_(95% bootstrap CI): 0.75(0.60,0.93)							
0.50	1.00	51	87	0.74(0.60,0.90)	−1	49	93	0.75(0.60,0.92)	0
0.40	0.96	66	89	0.74(0.60,0.90)	−1	64	92	0.75(0.60,0.92)	0
0.30	0.92	85	94	0.74(0.60,0.90)	−1	83	96	0.75(0.60,0.92)	0
0.20	0.89	110	99	0.71(0.59,0.80)	− 5	108	99	0.75(0.60,0.92)	0
0.10	0.86	150	89	0.73(0.60,0.86)	− 3	148	98	0.75(0.60,0.92)	0

^a^Overall survival;

^b^progression-free survival;

^c^one-sided significance level at stage 1;

^d^predefined threshold at stage 1;

^e^cumulative number of control arm events required at end of stage 1;

^f^percentages of trials that pass the interim stage and continue accrual to the final stage.

**Table 9:** Bootstrap results for the stopped trials based on ICON4 and RE01 in a two-stage design

		***I *and *D *outcomes are OS**^**a**^						***I *outcome is PFS**^**b**^**, *D *outcome is OS**^**a**^					
				**Estimated *HR***_***D ***_**for trials stopped at stage 1**						**Estimated *HR***_***D ***_**for trials stopped at stage 1**			
			**%**	**At interim point**		**After follow-up**				**At interim point**		**After follow-up**	
***α***_**1**_^**c**^	***δ***_**1**_^**d**^	***e***_**1**_^**e**^	**%Stop**	**Mean (centiles)**	**%Bias**	**Mean (centiles)**	**%Bias**	***e***_**1**_^**e**^	**%Stop**	**Mean (centiles)**	**%Bias**	**Mean (centiles)**	**%Bias**
a) ICON4 –		*HR*_*D*_(95% bootstrap CI): 0.82(0.67,0.99)											
0.50	1.00	75	1	1.05(1.00,1.20)	28	0.98(0.81,1.18)	20	75	1	1.17(0.80,1.66)	43	0.91(0.73,1.16)	11
0.40	0.96	98	3	1.01(0.97,1.18)	23	0.99(0.86,1.21)	21	97	1	0.99(0.71,1.31)	21	0.94(0.76,1.15)	15
0.30	0.94	126	4	0.98(0.94,1.12)	20	1.01(0.88,1.18)	23	125	2	0.87(0.66,1.12)	6	0.97(0.84,1.18)	18
0.20	0.91	163	8	0.96(0.91,1.09)	17	0.97(0.88,1.10)	18	162	5	0.89(0.70,1.12)	9	0.96(0.82,1.13)	17
0.10	0.89	222	14	0.99(0.89,1.55)	21	0.91(0.68,1.10)	11	221	12	0.86(0.70,1.05)	5	0.96(0.82,1.13)	17
b) RE01 –		*HR*_*D*_(95% bootstrap CI): 0.75(0.60,0.93)											
0.50	1.00	51	13	1.10(1.00,1.37)	47	0.96(0.76,1.21)	28	49	7	1.21(0.80,1.80)	61	1.11(0.77,1.56)	48
0.40	0.96	66	11	1.04(0.96,1.27)	39	0.91(0.74,1.16)	21	64	8	1.06(0.76,1.50)	41	0.97(0.69,1.37)	29
0.30	0.92	85	6	0.99(0.93,1.14)	32	0.92(0.77,1.14)	23	83	4	1.02(0.77,1.31)	36	0.91(0.71,1.22)	21
0.20	0.89	110	6	0.95(0.89,1.09)	27	0.90(0.77,1.06)	20	108	1	0.97(0.68,1.24)	29	0.93(0.69,1.23)	24
0.10	0.86	150	11	0.91(0.86,1.04)	21	0.88(0.72,0.95)	17	148	2	0.90(0.76,1.10)	20	0.86(0.73,1.05)	15

^a^Overall survival;

^b^progression-free survival;

^c^one-sided significance level at stage 1;

^d^predefined threshold at stage 1;

^e^cumulative number of control arm events required at end of stage 1.

**Figure 4:**
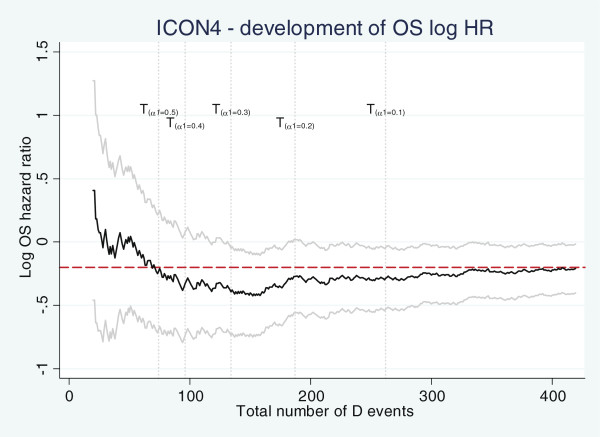
**Development of overall survival log hazard ratio over time in ICON4 trial.** Gray curves are the corresponding 95% CIs. The dashed horizontal line shows the overall (underlying) effect. The vertical dotted lines specify the interim analysis time points (see text for calendar time) where stage 1 analysis is carried out based on different design significance levels *α*_1_ – the *I* outcome is PFS.

In ICON4 and RE01, we also computed the average treatment effect for the stopped trials at the interim stage. The results in Table 9 reaffirm that follow-up decreases the amount of bias in most scenarios. It should be noted that unlike our simulation studies where (under the proportional hazards assumption) the treatment effect is assumed to be constant over time, in real trials the effect may not be constant over time. With real trial data we will not know whether the underlying process that created it satisfies the PH assumption or not. Even if the underlying data generating model did satisfy the PH assumption, it is still possible for a single realization of this process (that is one trial’s worth of data) to empirically depart from PH. In fact, as Figure 4 demonstrates the estimate of treatment effect in ICON4 fluctuates (in some parts markedly) early in the course of the trials. The final overall estimate for *HR*_*D*_is 0.82 - red dashed line. However, the mean bootstrapped estimate – that is the means of OS hazard ratios for all 5,000 replicated trials – changes from 0.83, 0.74, 0.70, 0.73 to 0.76 when *α*_1_ is 0.5, 0.4, 0.3, 0.2 and 0.1, respectively. The corresponding time points of interim analysis for these *α*_1_ values are 2.4, 2.9, 3.4, 4.1 and 4.9 years after the start of the trial, respectively. This is the reason for the (relatively large) bias in the average effect of stopped trials in some scenarios presented in Table 9. However, it can be argued that a larger bias in these situations is not so important since we are not claiming that the experimental treatment is effective.

## Discussion

In this paper, we have assessed the validity of the estimates of treatment effects resulting from a TAMS design, with a specific focus on bias. By defining the ‘selection bias’ in selected and dropped treatments, we have quantified its likely magnitude via a simulation study and bootstrap reanalysis of existing trials. Our results highlight that the amount of selection bias is generally small and its degree depends on the design parameters and the unknown true (underlying) effect values.

In the TAMS design, the bias generally tends to be larger when selecting ‘early’, that is, when the decision is based on a relatively small number of events. The results also show that, as pointed out by Royston *et al.*[5], under some assumptions bias in treatment effects on the definitive outcome can be markedly reduced by following all patients up to the planned end of the trial and performing analyses then, irrespective of whether recruitment was stopped early for lack of benefit. (Follow-up can also help in capturing the relevant information on safety endpoints.) Of course, it can be argued that by definition for arms that have stopped early a claim that the experimental treatment is better than the control is not made so the fact that treatment effect is biased is less important.

In our analyses, by choosing different significance levels for the first interim stage we also explored the timing of the first interim stage analysis at which the bias will be small. Our investigations suggest that the bias will be minimal, if the first interim stage is placed at a significance level of 0.3 or less. As a trade-off between the amount of bias and efficiency, we suggest that the first interim stage to be defined by a significance level between 0.2 and 0.3. This suggestion accords with the recommendations made by Barthel *et al.*[14]. However, this is only a practical recommendation with respect to bias and does not reflect an optimal design which could be obtained from a simulation study or theoretical calculation. Furthermore, we have shown that the bias in the treatment effect would become negligible if the TAMS trials were powered at small effect sizes investigating treatments with true large effect sizes. This, however, would in practice increase the number of events required and so the cost and duration of the trial. Finally, our simulation results showed that using an intermediate outcome measure reduces the selection bias in the estimates of treatment effects in both selected and dropped arms – provided that the chosen intermediate outcome measure satisfies the conditions set out by Royston *et al*. [1].

However, we emphasize that the selection bias in the estimate of treatment effect of trials that reach the final stage is a more major consideration than that in stopped trials. An effective experimental arm is very likely to reach the final stage of a TAMS trial, and the results of such trials are more likely to be adopted into clinical practice. Our empirical studies showed that the size of selection bias for the trials that reach the final stage is generally small. In fact, the bias is negligible if the experimental arm is truly effective.

For a dropped treatment arm, the estimate of the treatment effect is generally on the extremes of its sampling distribution – see Figure 2 and also Figure 2 in [8]. The estimate, as suggested by Goodman [16] and Freidlin and Korn [17], is generally on a random high (or low, depending on the direction of efficacy). Freidlin and Korn [17] argued that one should take this into consideration, and compare the average effect in the dropped arm with the average effect of a ‘similar’ fixed sample size trial, which is on the random high – see [16,17] for their definition of ‘similar’. Their proposed fixed sample size comparator is hypothetical and quite complicated. In our simulation studies, we also compared the average effect in the dropped arm of a TAMS design with their proposed comparator (results not shown). Our findings showed that after the follow-up the average effect in the dropped arm is almost identical to their proposed comparator. Freidlin and Korn [17] concluded that in trials with a well-designed interim-monitoring plan, the selection bias is negligible if one compares the average effect in the dropped arms to their fixed sample size comparator. Therefore, our conclusions about the TAMS designs, although in a slightly different context, agree in principle with the findings of Freidlin and Korn [17]’s investigations.

Several unbiased estimators of the treatment effect have been proposed to correct for bias inherent in two-stage designs of the TAMS type, although they were originally developed in a different context for trials with continuous, conditionally normal outcome variables. Cohen and Sackrowitz [18] and Bowden and Glimm [19]’s formula can be applied to the definitive endpoint at the end of a two-stage trial when the definitive endpoint has been used to decide on continuing/dropping the research arm at the interim analysis. Sill and Sampson [20] extended Cohen and Sackrowitz’s unbiased (UMVCUE) estimator to the case where the interim decision is based on an intermediate outcome. We chose not to include a thorough comparison of these bias-adjusted estimators in our paper for several reasons. First and foremost, we are dealing with (censored) time-to-event data and Sill and Sampson’s [20] formulae do not naturally extend to such a case. Second, in our situation the bias in the standard treatment effect estimates at the end of the trial was shown to be small. Third, the aforementioned formulae are presently only available for two-stage trials and are inapplicable to TAMS designs with more than two stages. This is a topic for further research. Finally, even if an unbiased estimator was available, it might not be preferred to the slightly biased standard (ML) estimator because its mean square error is likely to be larger [20,21].

## Conclusions

Our empirical studies show that the estimated treatment effect on the definitive outcome has a small bias at the time of ceasing recruitment to an arm. However, if follow-up is continued to the planned end of the trial, even this small bias decreases markedly. Our results also suggest that in trials with a truly efficacious experimental arm that continue to the planned end, the bias is very small and of no practical importance.

## Abbreviations

HR: hazard ratio; MPLE: maximum partial likelihood estimate; OS: overall survival; PFS: progression-free survival; PH: proportional hazards.

## Competing interests

The authors declare that they have no competing interests.

## Authors’ contributions

BCO carried out the analysis and drafted the manuscript. MP, PR and JB helped to draft the manuscript and were involved in the discussion regarding the analysis methods. All authors read and approved the final manuscript.

## Authors’ information

BCO is a medical statistician in the Hub for Trials Methodology Research at the MRC Clinical Trials Unit with a particular interest in clinical trials methodology. MP is the director of the MRC Clinical Trials Unit with a wide-ranging interest in the design, analysis and conduct of randomized controlled clinical trials, as well as being involved in statistical methodology. PR is a senior statistician with 30 years of experience, with a strong interest in biostatistical methods and in statistical computing and algorithms. JB is a medical statistician in the Hub for Trials Methodology Research at the MRC Biostatistics Unit with a particular interest in modelling, understanding and correcting for selection bias in medical data.
